# Modulation of Preactivation of PPAR-**β** on Memory and Learning Dysfunction and Inflammatory Response in the Hippocampus in Rats Exposed to Global Cerebral Ischemia/Reperfusion

**DOI:** 10.1155/2012/209794

**Published:** 2012-09-27

**Authors:** Ge Kuang, Qin He, Yunmei Zhang, Ruichun Zhuang, Anling Xiang, Qingsong Jiang, Ying Luo, Junqing Yang

**Affiliations:** ^1^Department of Pharmacology, The College of Pharmacy, Chongqing Medical University, Chongqing 400010, China; ^2^Department of Hepatobiliary Surgery, The First Affiliated Hospital, Chongqing Medical University, Chongqing 400010, China; ^3^Department of Nursing, Nursing School of Chongqing Medical University, Chongqing 400010, China

## Abstract

The aim of this study is to investigate the neuroprotective effects and relevant mechanism of GW0742, an agonist of PPAR-**β**, after global cerebral ischemia-reperfusion injury (GCIRI) in rats. The rats showed memory and cognitive impairment and cytomorphological change in the hippocampus neurons following GCIRI. These effects were significantly improved by pretreatment with GW0742 in the dose-dependent manner. The expressions of IL-1**β**, IL-6, and TNF-**α** were increased after GCIRI, while the increases in these proinflammatory cytokines by GCIRI were inhibited by GW0742 pretreatment. Similarly, GW0742 pretreatment also improved the GCIRI-induced decrease in the expression of IL-10, which can act as an inhibitory cytokine to reduce cerebral ischemic injury. For another, NF-**κ**B p65 expression was significantly increased in hippocampal neurons with apparent nuclear translocation after global cerebral IRI, and these phenomena were also largely attenuated by GW0742 pretreatment. Moreover, the mRNA and protein expressions of PPAR-**β** were significantly decreased in GCIRI + GW0742 groups when compared with those in GCIRI group. Our data suggests that the PPAR-**β** agonist GW0742 can exert significant neuroprotective effect against GCIRI in rats via PPAR-**β** activation and its anti-inflammation effect mediated by the inhibition of expression and activation of NF-**κ**B in the hippocampus.

## 1. Introduction

Global cerebral ischemia-reperfusion injury (GCIRI) can occur in patients who are successfully resuscitated from various clinical conditions like cardiac arrest, shock, and asphyxia [1] and are characterized by a deterioration of ischemic but salvageable brain tissue after reperfusion [2]. Many of these patients can suffer from various degrees of memory problem and learning dysfunction [3], suggesting an impairment in the hippocampus which is a primary region of the brain controlling the formation of memories and learned behaviors [4]. It was reported that hippocampus, especially CA1 area, was selectively vulnerable during global cerebral ischemia [5, 6]. The memory and cognitive problem caused by cerebral IRI tremendously impacts on the patients' life [3]. However, effective approaches to prevent and treat this memory and cognitive impairment following global cerebral ischemia-associated events are still lacking.

A number of studies have demonstrated cerebral ischemia is frequently accompanied by inflammation, and reperfusion after brain ischemia increases the inflammatory reactions, which can worsen neuronal injury [7–9]. Proinflammatory cytokines, such as tissue necrosis factor-*α* (TNF-*α*), interleukin (IL)-1, and IL-6, have been implicated as important mediators of ischemia/reperfusion injury following both focal and global cerebral ischemia [9–11]. In other words, they contribute to pathogenesis, exacerbating ischemic-reperfusion brain tissue damage [12, 13]. Specifically, a substantial and persistent production of these cytokines can significantly increase the risk and extent of brain injury [10, 14]. For another, NF-*κ*B, an important transcription factor that plays a pivotal role in mediating inflammatory response to proinflammatory cytokines [15], was also upregulated during cerebral IRI [16]. Similar cerebral IRI-induced inflammation reaction and neural damage were reportedly observed in the hippocampus [17, 18].

Peroxisome proliferator-activated receptors (PPARs) are members of the steroid hormone nuclear receptor family with three isotypes in mammals: PPAR-*α*, PPAR-*β* (also called PPAR-*δ*), and PPAR-*γ* [19]. In contrast to PPAR-*α* and PPAR-*γ* that are preferentially expressed in liver and kidney, and adipose tissues, respectively, PPAR-*β* is expressed in most tissues [20], especially found throughout the brain, with particularly high levels in some regions including the hippocampus [21]. Intriguingly, recent reports have shown that PPAR-*β* has a neuroprotective effect on the central nervous system. To investigate the role of PPAR-*β* in focal cerebral ischemia damage, some studies [22, 23] found that, compared with wild type, PPAR-*β*-null mice exhibited a significant increase in the infarct size. Reciprocally, central administration of PPAR-*β* agonists significantly and concentration-dependently attenuated the ischemic brain damage after reperfusion in rats [24]. Moreover, the use of PPAR-*β* agonists in preclinical studies suggests that PPAR-*β* also possesses anti-inflammatory properties [25]. However, it is unknown whether PPAR-*β* can exert beneficial action on the memory and learning function and improve inflammatory reaction in the hippocampus after GCIRI. Therefore, in this study we use GCIRI animal modal to test whether: (1) preactivation of PPAR-*β* could prevent or improve the impairment of learning and memory caused by IRI; (2) the expression of PPAR-*β* in the hippocampus increased after IRI; (3) PPAR-*β* agonists could improve the IRI-induced inflammation reaction and cytomorphological change in the hippocampus.

## 2. Materials and Methods

### 2.1. Animals

60 pathogen-free Sprague-Dawley male rats (200–250 g) were used in this study. The experimental protocols were conducted in strict accordance with the Guide for the Care and Use of Laboratory Animals and were approved by the Animal Care and Use Committee at Chongqing Medical University.

### 2.2. GCIRI Animal Modal

Global cerebral ischemia was induced by ligation of the bilateral common carotid artery combined with hemorrhagic hypotension in rats [26, 27]. Briefly, 30 min after administration of GW0742, rats were anesthetized with chloral hydrate (10 mL/kg IP) and then bilateral common carotid arteries and the right common jugular vein were dissected. The right common jugular vein was ligated at the distal end and then intubated. 30% of total blood volume of the rats was extracted following 2 mL heparinized saline infusion (250 U heparin per 100 mL saline). And then bilateral common carotid arteries were occluded with bulldog clamps for 20 min. The extracted blood was then infused back into the rats and the right common jugular vein was ligated.

### 2.3. Microinjection into the Lateral Ventricle

GW0742 was dissolved in 30% DMSO to three concentrations of 18 mg/mL, 6 mg/mL, and 2 mg/mL, respectively. 30% DMSO as vehicle was not found in any neuronal injury and behavioral function impairment in our pilot study. Then 10 *μ*L of the solutions with the dosage of 20 *μ*g/rat, 60 *μ*g/rat, and 180 *μ*g/rat were administered by intracerebroventricular (I.C.V.) infusion into the right lateral ventricle 30 min before bilateral common carotid artery occlusion (BCCAO). BCCAO was performed similar to the previous report [24] with some slight modification. Briefly, rats were anesthetized with chloral hydrate (10 mL/kg, IP) and fixed in a stereotaxic apparatus with the hairs in the vertex shaved. A midline incision was made in the vertex. The skull was drilled at the following coordinates: 0.8 mm posterior to the bregma and 1.5 mm lateral to the midsagittal suture. Animals were chronically implanted with a 25 *μ*L microinjector fixed 4.0 mm ventral to the skull and were infused with the GW0742 solution or vehicle slowly into the right lateral ventricle within 4 min.

### 2.4. Test of Spatial Learning and Memory Function with Morris Water Maze Test

Spatial learning and memory function of the rat was tested by Morris water maze with tap water (21 ± 2°C) 7 days after I/R [28] for 5 days (i.e., day 8–day 12 post-GCIRI). The test was divided into two phases: Phase I was the training phase and Phase II was the testing phase. At phase I, rats (*n* = 12 from each group) were trained for 4 consecutive days (i.e., day 8–day 11 post-GCIRI), with 4 trials on each day at 20:00 pm–23:00 pm. During each trial, individual rat was put in the pool from A, B, C, and D quadrant separately. Each rat was given 180 seconds (s) to search for and locate the submerged platform. After arriving at the platform, the animal was allowed to stay on it for 10 s. The latency time to find the platform was recorded. If a rat failed to locate the platform within 180 s, it would be guided gently to the platform and the latency time was recorded as 180 s. The average time from 4 trials represented as the daily result for the animal. At phase II, animals were tested for spatial learning and memory function on day 12 post-GCIRI. Individual rat was put from one of A, B, C, and D quadrant randomly in the pool, and the latency time was recorded as the result of spatial memory function test. All of the ischemic animals had no motor damage. 

### 2.5. Histopathology and Immunohistochemistry

On the twelfth day after GCIRI, the rats were anesthetized with 4% chloral hydrate (400 mg/kg, IP), and brains were then transcardially perfuse with 4% paraformaldehyde. Paraffin sections (5 *μ*m thick) of coronal slices of the dorsal hippocampal CA1 subfield were made in each rat and stained with hematoxylin and eosin (HE). For assessment of cell counts from H&E stained sections, 10 consecutive high power fields were sampled from the dorsal hippocampal CA1 subfield. Pyramidal cells with a distinct nucleus and nucleolus were regarded as intact neurons. Counts of intact neurons were performed from the ischemic and sham brains using a microscope at 400x magnification. The extent of cell death was estimated by numbers of intact cells from the sham minus numbers from the ischemic brain and divided by counts from the shams. An immunohistochemical method was used to test the expression of NF-*κ*B p65 protein. The LAB-SA kit with the polyclonal antibody for NF-*κ*B p65 protein (1 : 50, Santa Cruz) was used according to the manufacture's directions. For NF-*κ*B stains, all cells in hippocampal CA1 on two coronal sections were counted, and then numbers of cells with nuclear staining were counted separately. The percentage of cells with nuclear staining was estimated by their numbers divided by the total cell numbers. All histological assessments were made by an inspector blinded to the conditions of the experiments.

### 2.6. RT-PCR

Reverse transcription-polymerase chain reaction (RT-PCR) was used to measure mRNA expression of PPAR-*β*.

### 2.7. Primers Design

The primers of PPAR-*β* were designed on the basis of the rat PPAR-*β* cDNA sequence in Genebank using Primer Premier 5.0 (Premier Biosoft International, Palo Alto, California, USA) and synthesized by Sangon Biotech Co., Ltd. (Shanghai, China) as follows: forward, 5′-GCCGCCCTACAACGAGATCA-3′; reverse, 5′-CCACCAGCAGTCCGTCTTTGT-3′. The PCR product length was 143 bp. The primers of endogenous *β*-Actin were purchased from Dingguo Biotech incorporated company (Beijing, China) with the sequences as follows: forward: 5′-GTGGGGCGCCCCAGGCACCA-3′; reverse, 5′-CTTCCTTAATGTCACGCACGATTTC-3′. The PCR product length was 540 bps. 

### 2.8. Isolation of Total RNA

Total RNA from tissue homogenate of the rat hippocampus was prepared with the use of Biozol reagent from BioFlux, Inc. In brief, cells were lysed and extracted in an RNase free homogenizer by adding 1 mL of Biozol. The lysate was added to 200 *μ*L of chloroform, and the solution was mixed and centrifuged. The supernatant was removed, mixed with an equal volume of isopropanol, and kept at −20°C for at least 20 minutes. After centrifugation at 12000 g for 10 minutes at 4°C, the sediment was washed with 75% ethanol and then centrifuged again for 5 minutes at 4°C. The RNA fraction was then resuspended in sterile water treated with 1% DEPC. Total RNA was quantified by spectrophotometry and then adjusted to concentrations of 1 *μ*g/*μ*L.

### 2.9. Synthesis of cDNA by Reverse Transcription

cDNA was synthesized in a 20 *μ*L reaction volume containing 1 *μ*g total RNA, 1 *μ*mol/L oligo (dT) and 0.2 mmol/L dNTPs (Qiagen), 10 U of RNasin (Promega, Madison, WI), and 4 U of ReverTra Ace (TOYOBO) for 20 min at 42°C, then 5 min at 99°C, and 5 min at 4°C. cDNA synthesized was preserved at −20°C.

### 2.10. PCR

cDNA was amplified in a system with 0.2 m mol/L dNTPs, 1 *μ*mol/L of each primer, 2 mmol/L MgCl_2_, and 2.5 U *Taq* polymerase (Promega). PCR was performed for 35 cycles alternating between 94°C for 4 min, and then 94°C for 15 seconds and 57°C for 15 seconds for PPAR-*β*, followed by extension at 72°C for 5 minute. The amplified products were analyzed on 2% agarose gel. The relative mRNA level of PPAR-*β* was normalized to endogenous *β*-actin mRNA for each sample.

### 2.11. Western Blot Analysis

Western-blotting was used to measure protein expression of PPAR-*β*. Hippocampal tissue added with 0.5 mL of tissue lysis solution was homogenized and then lysed by chip sonication for 3–5 min at 4°C. The lysate was centrifuged. The supernatant was collected and quantified for total protein. Samples (20 *μ*g of protein) were electrophoresed onto a 5–10% SDS/polyacrylamide gel (SDS/PAGE) and transferred to PVDF membranes. The membranes were blocked in TBST buffer containing 20 mM Tris-HCl, 5% nonfat milk, 150 mM NaCl, and 0.05% Tween-20 (pH 7.5) for 1 h at room temperature (RT). Thereafter, the blot was washed with PBST 3 times at 10 min intervals, incubated with primary rabbit anti-rat PPAR-*β* antibody (1 : 1000; Santa Cruz, CA) overnight at 4°C. The membrane was washed with PBST 3 times at 10 min intervals, incubated with the secondary antibody (1 : 5000; anti-rabbit IgG conjugated with horse radish peroxidase, Santa Cruz, CA) at RT for 1 h, and then washed three times each at 10 min intervals with PBST. The color reaction was developed by the ECL System (Pierce) in Bio-Rad imaging system. The relative protein levels of PPAR-*β* were normalized to endogenous *β*-actin protein for each sample.

### 2.12. Measurement of TNF-*α*, IL-1*β*, IL-6, IL-10

Hippocampal tissues added with saline by weight-volume ratio 1 : 9 were homogenized, and centrifugated and the supernatant were collected for test. Rat TNF-*α*, IL-1*β*, IL-6, and IL-10 in the tissue homogenate of hippocampus were measured by ELISA. The ELISA kit (ADI, Stamford, USA) with the monoclonal antibody was used according to the manufacture's directions. 

### 2.13. Reagents

GW0742 was purchased from Sigma-Aldrich Chemical Co. (St. Louis, MO, USA.). TNF-*α*, IL-1*β*, IL-6, and IL-10 ELISA Kit were purchased from American Diagnostica Inc. (ADI, Stamford, USA). RNAlater was purchased from QIAGEN Sample & Assay Technologies (Dusseldorf, German). BIOZOL was purchased from BioFlux (Tokyo, Japan). ReverTra Ace-*α* was purchased from TOYOBO CO., LTD (Osaka, Japan). Taq DNA Polymerase was purchased from Promega Corporation (Madison, WI, USA). *Pierce ECL* Western Blotting Substrate (32106) was purchased from Pierce Biotechnology (USA). Rabbit anti-rat monoclonal antibody against PPAR-*β*, rabbit anti-rat polyclonal antibody of NF-*κ*B p65, and horseradish peroxidase-conjugated goat anti-rat secondary antibodies were purchased from Santa Cruz Biotechnology, Inc. (Santa Cruz, CA, USA). 

### 2.14. Experiment Protocol

Animals were randomly allocated to the following groups.

1. Vehicle Sham Group. The animals were subjected to the same surgical procedures as other groups except for exsanguinations from right common jugular vein and occlusion of the bilateral common carotid arteries with vehicle injected into the lateral ventricle (*n* = 12).
2. Vehicle Model Group. The animals were subjected to GCIRI with vehicle injected into the lateral ventricle (*n* = 12).
3. GW0742-20 Model Group. Identical to vehicle model group except for receiving 20 *μ*g GW0742 microinjection into the lateral ventricle (*n* = 12).
4. GW0742-60 Model Group. Identical to vehicle model group except for receiving 60 *μ*g GW0742 microinjection into the lateral ventricle (*n* = 12).
5. GW0742-180 Model Group. Identical to vehicle model group except for receiving 180 *μ*g GW0742 microinjection into the lateral ventricle (*n* = 12).

All animals were allowed to recover from day 1-day 7 and were tested for spatial learning and memory function 7 days after GCIRI. Animals received special memory and learning training for 4 consecutive days (i.e., from day 8 to day 11 post-GCIRI). On day 12 animals were tested for spatial learning and memory function and then euthanized for histopathology test (*n* = 3), biochemistry test (*n* = 5), and RT-PCR and western-blotting (*n* = 4).

### 2.15. Statistical Analysis

Data are presented as mean ± SD analyzed by SPSS12.0. Differences between groups were analyzed with the one way ANOVA. Values of *P* < 0.05 were regarded as statistically significant.

## 3. Results

### 3.1. Effect of GW0742 on Spatial Learning and Memory Function of Rats after GCIRI

As shown in Table 1, at phase 1, the exploring time in all of the groups tended to decrease over time and it was significantly longer in the vehicle model groups than in the sham group for all the testing days (*P* < 0.01), suggesting that the vehicle model group needed a significantly longer time compared with the sham group to find the platform. No significant difference for the exploring time was found between GW0742-20 model group and vehicle model group (*P* > 0.05). Compared with vehicle model group, GW0742-60 model group and GW0742-180 model group generally presented a remarkable shorter exploring time (*P* < 0.05) with the latter group presenting a shorter but no significant different in exploring time than the former group. Compared with GW0742-20 model group, GW0742-180 model group exhibited a significant shorter exploring time on day 11. At phase 2 (i.e., on the twelfth day), the exploring time for the vehicle model group to find the platform increases significantly compared with the vehicle sham group, but GW0742 60 *μ*g and the GW0742 180 *μ*g inhibit the increase of the exploring time of GCIRI rats significantly with the latter group presenting a shorter but no significant different in exploring time than the former group; whereas, GW0742 20 *μ*g shows little effect on the increase of the escape latency in rats with GCIRI (Table 1). Compared with GW0742-20 model group, GW0742-180 model group exhibited a significant shorter exploring time on day 12 (*P* < 0.05).

### 3.2. Effect of GW0742 on Morphologic Changes of Hippocampal Neurons of Rats after GCIRI

**Figure 1:**
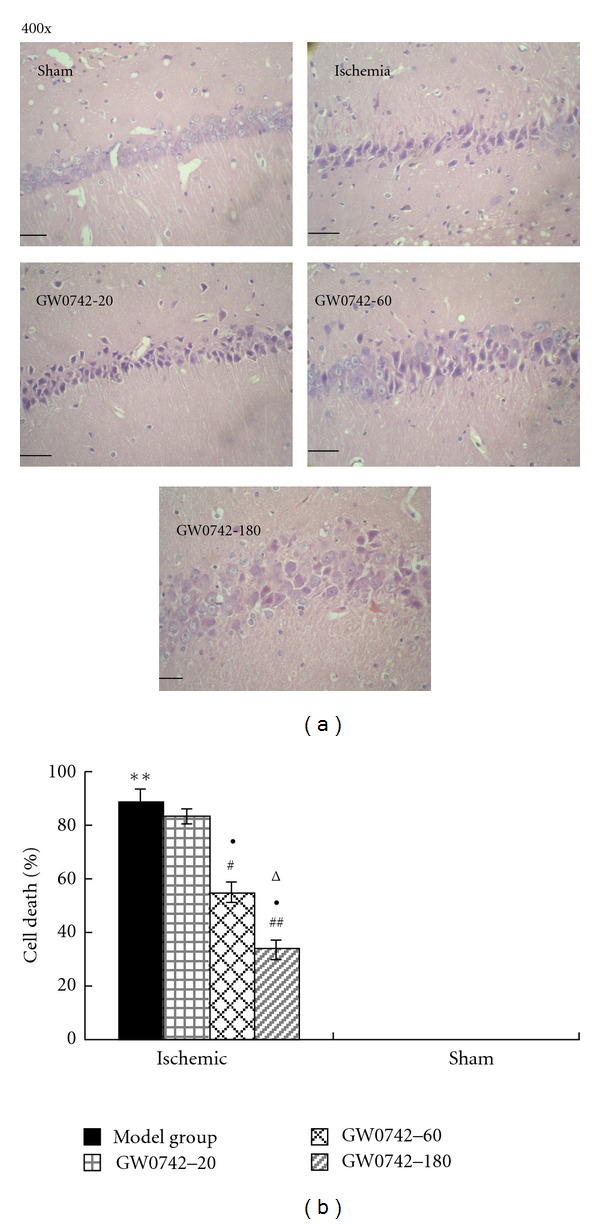
PPAR-*β* agonist GW0742 improves the global cerebral IRI-induced cytomorphological change in the hippocampus. (a) Representative pictures of H&E stained CA1 sections on day 12 post-GCIRI shown at 400x magnification. Scale bars = 50 *μ*m. (b) Group data showing the effect of GW0742 on the cell death rate. **P < 0.01 compared with vehicle sham group; ^#^ *P* < 0.05, ^##^ *P* < 0.01 compared with the vehicle model group; ^Δ^ *P* < 0.05 compared with GW0742 20 model group; ^●^ *P* < 0.05 compared with GW0742 60 model group, (*n* = 3).

**Figure 2:**
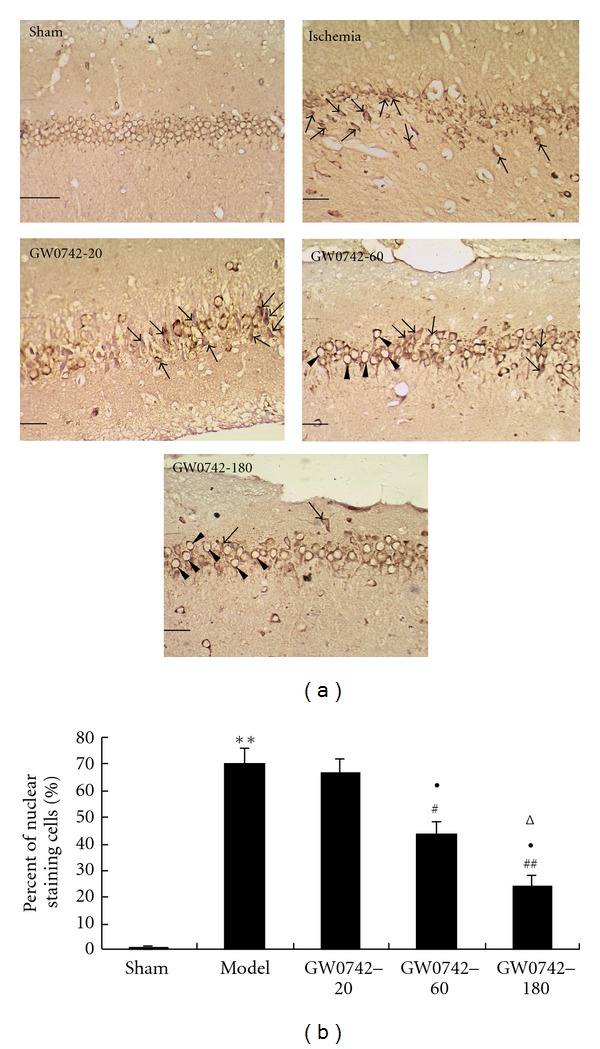
GW0742 inhibits the GCIRI-induced expression and nuclear translocation of NF-*κ*B in the hippocampus. (a) Representative images of the hippocampal CA1 region after GCIRI. Once NF-*κ*B is activated, it translocates into the nucleus. In the vehicle sham group (Sham), weak cytosolic staining was observed, but no obvious nuclear staining was seen. In the vehicle model animals (Ischemia) and the GW0742-20 model group (GW0742-20), NF-*κ*B staining was significantly increased and was seen in the nucleus in most of cells, see the arrow-pointed cells and cytosol. In the GW0742-60 model group (GW0742-60), cells with nuclear NF-*κ*B staining were deceased compared with the vehicle model group. In the GW0742-180 model group (GW0742-60), staining was mostly confined to the cytosol (see the arrow head-pointed cells) with very faint staining in the nucleus (scale bars = 50 *μ*m). (b) Group data showing the effect of GW0742 on NF-*κ*B expression and translocation. **P < 0.01 compared with vehicle sham group; ^#^ *P* < 0.05, ^##^ *P* < 0.01 compared with vehicle model group; ^Δ^ *P* < 0.05 compared with GW0742-20 model group; ^●^ *P* < 0.05 compared with GW0742 60 model group, (*n* = 3).

No cell death occurred in the CA1 region of dorsal hippocampus in the vehicle sham group. On the contrary, significant (~90%) pyramidal cell death companied with remarkable cell loss occurred in the vehicle model group (*P* < 0.01 compared with the vehicle sham group). Dead and dying pyramidal cells in the injured hippocampi displayed cellular necrosis, characterized by pyknotic nuclei and irregular cell contours. There were still a large number of neurons with nuclear pyknosis in GW0742-20 group, with no significant difference compared with the vehicle model group. However, the hippocampal CA1 pyramidal cell loss and nuclear pyknosis were significantly diminished in GW0742-60 model group and GW0742-180 model group (especially in the latter group), relative to the vehicle model group and GW0742-20 group (Figure 1(a)). Quantification of remaining, viable neurons showed that compared with the vehicle model group, GW0742-60 model group (*P* < 0.05) and GW0742-180 (*P* < 0.01) model group exhibited 38.1% and 61.8% reduction in cell death, respectively (Figure 1(b)). There was no significant difference between the GW0742-20 model group and the vehicle model group for the cell death rate (*P* > 0.05). And there were significant differences in GW0742-60 model group and GW0742-180 model group versus with GW0742-20 model group and in GW0742-180 model group versus with GW0742-60 model group (*P* < 0.05, Figure 2(b)).

### 3.3. Effect of GW0742 on NF-*κ*B p65 Expression in Hippocampus of Rats after GCIRI

Immunochemical results for NF-*κ*B's p65 subunit were showed in Figure 2(a). In the vehicle sham group, weak cytosolic staining was observed, and there was virtually no NF-*κ*B immunoreactivity in the nucleus. NF-*κ*B staining intensity was significantly increased both in the nucleus and cytoplasm (over 60% cells) in the vehicle model animals (*P* < 0.01 compared with the vehicle sham group). However, compared with the vehicle model group NF-*κ*B staining intensity did not change in the GW0742-20 model group, but significantly decreased in the GW0742-60 and GW0742-180 model group (especially in GW0742-180 model group). Percentages of cells with p65 immunoreactivity were increased in the vehicle model animals, but this increase was significantly attenuated in the GW0742-60 and GW0742-180 model group. In other words, percentages of cells with nuclear p65 staining revealed significantly lower percent in the GW0742-60 and GW0742-180 model group compared with the vehicle model group (*P* < 0.05 and *P* < 0.01, resp.). There was no significant difference for percentages of cells with nuclear p65 staining between the GW0742-20 model group and the vehicle model group (*P* > 0.05). And there were significant differences in GW0742-60 model group and GW0742-180 model group versus with GW0742-20 model group and in GW0742-180 model group versus with GW0742-60 model group (*P* < 0.05, Figure 2(b)).

### 3.4. Effect of GW0742 on PPAR-*β* mRNA Expression in Hippocampus of Rats after GCIRI

**Figure 3:**
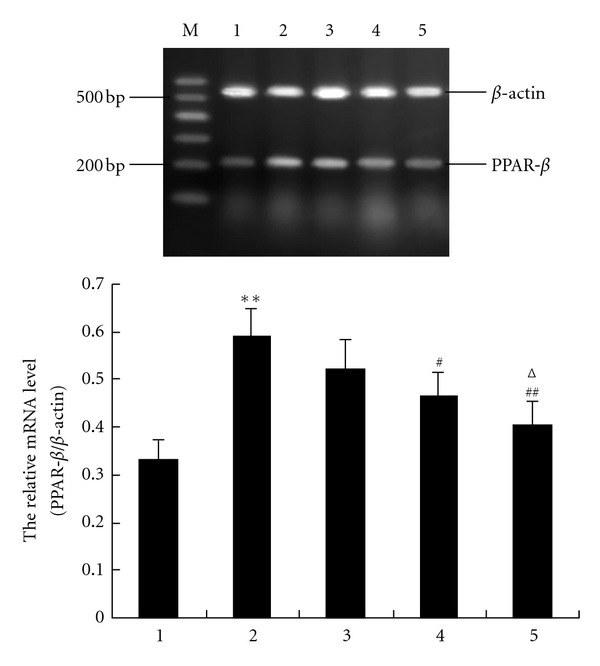
GW0742 inhibits the GCIRI-induced increase of mRNA expression of PPAR-*β* in hippocampus of rats (*n* = 4). The relative mRNA level of PPAR-*β* was normalized to endogenous *β*-actin mRNA for each sample. M: DNA marker, 1: vehicle sham group, 2: vehicle model group, 3: GW0742 20 model group, 4: GW0742 60 model group, and 5: GW0742 180 model group. Dates are expressed as mean ± SD of four individual experiments. **P < 0.01 compared with vehicle sham group; ^#^ *P* < 0.05, ^##^ *P* < 0.01 compared with vehicle model group; ^Δ^ *P* < 0.05 compared with GW0742 20 model group.

As shown in Figure 3, PPAR-*β* mRNA expression in hippocampus of vehicle model group increased significantly compared with that of the sham group (*P* < 0.01) with a rise rate of 78.55%. GW0742 60 *μ*g and GW0742 180 *μ*g i.c.v. infusion significantly inhibited the increase of the PPAR-*β* mRNA expression (*P* < 0.05 and *P* < 0.01, resp.) with an inhibition rate of 20.98% and 31.30%, respectively. No significant difference was observed between the GW0742 20 model group and the vehicle model group (*P* > 0.05). 

### 3.5. Effect of GW0742 on PPAR-*β* Protein Expression in Hippocampus of Rats after GCIRI

**Figure 4:**
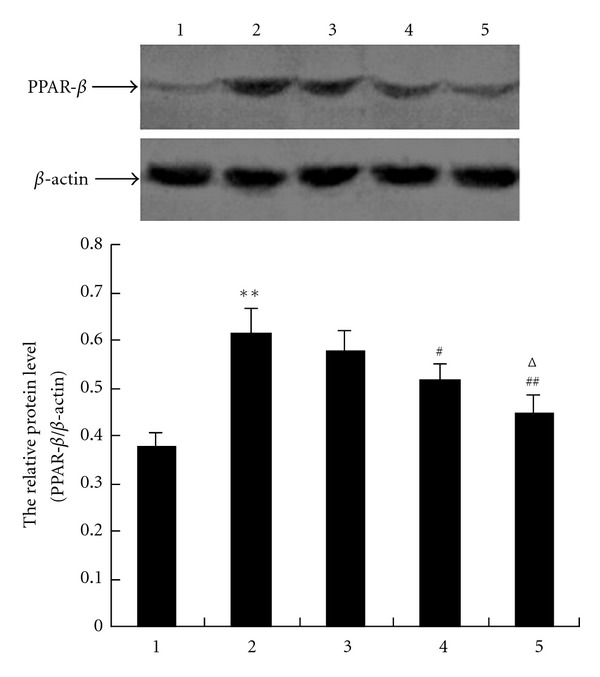
GW0742 inhibits protein expression of PPAR-*β* in hippocampus of rats after GCIRI (*n* = 4). The relative protein level of PPAR-*β* was normalized to endogenous *β*-actin protein for each sample. 1: vehicle sham group, 2: vehicle model group, 3: GW0742 20 model group, 4: GW0742 60 model group, and 5: GW0742 180 model group. Dates are expressed as mean ± SD of four individual experiments. **P < 0.01 compared with vehicle sham group; ^#^ *P* < 0.05,  ^##^ *P* < 0.01 compared with vehicle model group; ^Δ^ *P* < 0.05 compared with GW0742 20 model group.

Similar to the PPAR-*β* mRNA expression, PPAR-*β* protein expression in hippocampus of the vehicle model group increased significantly compared with that of the vehicle sham group (*P* < 0.01) with the rise rate of 63.20%. Hippocampus PPAR-*β* protein expression of GW0742 60 group (*P* < 0.05) and GW0742 180 group (*P* < 0.01) decreased obviously compared with the vehicle model group, and their inhibition rates were 15.69% and 27.29%, respectively. No significant difference was observed between the GW0742 20 model group and the vehicle model group (*P* > 0.05, Figure 4).

### 3.6. Effect of GW0742 on the Level of Inflammatory Factors in Hippocampus of Rats after GCIRI

Compared with the vehicle sham group, the vehicle model group exhibited a higher level of TNF-*α*, IL-1*β*, and IL-6 but a lower level of IL-10 in hippocampus (*P* < 0.01). GW0742 administration i.c.v. dose-dependently inhibited the increase of TNF-*α*, IL-1*β*, and IL-6 and the reduction of IL-10 in hippocampus of cerebral ischemia-reperfusion rat (Table 2).

## 4. Discussion

In this study, we studied the protective effect of the PPAR-*β* agonist GW0742 on learning and memory function and inflammatory response in the hippocampus in rats exposed to global cerebral ischemia/reperfusion. We found after GCIRI that (1) preactivation of PPAR-*β* could improve the impairment of learning and memory; (2) the expression of PPAR-*β* in the hippocampus increased, as was dose-dependently attenuated by pretreatment with GW0742; (3) PPAR-*β* agonists could improve the IRI-induced inflammation reaction and cytomorphological change in the hippocampus, and (4) the anti-inflammation effect of PPAR-*β* was mediated by inhibition of the expression and activation of NF-*κ*B in the hippocampus. 

The hippocampus is one of the brain areas most sensitive to ischemia/reperfusion [5, 6]. The majority of *in vivo* models of hippocampus ischemia rely on middle cerebral arteries occlusion predominantly affecting the region [29–31]. However, this focal cerebral IRI model cannot reflect the true situation in patients with cardiac arrest where global cerebral blood flow is interrupted [1]. Furthermore, the most common model of global cerebral IRI model is achieved by bilateral occlusion of the common carotid arteries [32, 33], but it is a model of incomplete global cerebral ischemia with only 50% decrease in cerebral blood flow in rats [34]. In our study we used bilateral occlusion of the common carotid arteries plus hemorrhagic hypotension to more closely mimic the clinical conditions of global cerebral ischemia. With this model, our results showed that transient cerebral ischemia/reperfusion caused a significant compromise of spatial learning and memory function, accompanied with a marked inflammatory reaction and a remarkable cytomorphological change in the hippocampus. All these *in vivo* and *in vitro* results demonstrate an impairment of the hippocampus, offering the feasibility for us to further explore the neuroprotective effect of PPAR-*β* on the hippocampus of rats exposed to GCIRI. 

PPAR-*β* is one of the three members of PPARs and is expressed throughout the brain, with high levels in the hippocampus [21]. Emerging studies have reported the neuroprotective effects of PPAR-*β* in animal models of brain damage induced by cerebral transient focal ischemia. For example, compared with wild type, PPAR-*β*-null mice exhibited a significant increase in the infarct size [22, 23], while central administration of PPAR-*β* agonists significantly and dose-dependently attenuated the ischemic brain damage after reperfusion in rats [24, 35, 36]. In agreement with these studies, our data showed that PPAR-*β* agonist GW0742 could dose-dependently attenuate the increase in the escape latency to find the platform in the rats exposed to GCIRI, suggesting an improvement in spatial learning and memory dysfunction. Interestingly, the PPAR-*β* mRNA and protein expression in the hippocampus markedly increased after IRI, as was dose-dependently attenuated by pre-treatment with GW0742 administered in the lateral ventricle. These results suggest that not only PPAR-*β* can exhibit neuroprotective effect on the hippocampus function after GCIRI, but also its expression level in the hippocampus may predict the damage degree by GCIRI of this major brain region controlling the formation of memory and learning [4]. 

Numerous studies have demonstrated that cerebral ischemia is commonly accompanied by inflammation, and reperfusion after brain ischemia increases the inflammatory reactions, which can worsen neuronal injury [7–9]. Proinflammatory cytokines, such as tissue necrosis factor-*α* (TNF-*α*), interleukin (IL)-1*β*, and IL-6 have been implicated as important mediators of ischemia/reperfusion injury following both focal and global cerebral ischemia [9, 10, 12]. Specifically, a substantial and persistent production of these cytokines can significantly increase the risk and extent of brain injury [10, 11, 37]. For another, NF-*κ*B, an important transcription factor that plays a pivotal role in mediating inflammatory response to proinflammatory cytokines [15] was also upregulated during cerebral IRI [16]. Similar cerebral IR-induced inflammation reaction and neural damage were reportedly observed in the hippocampus [17, 18]. These results demonstrate that inflammatory reaction contributes to pathogenesis of exacerbating ischemic-reperfusion brain tissue damage [12, 13]. Recent studies found PPAR-*β* processed anti-inflammation activity, especially with an ability to regulate central inflammation in the damaged brain [25, 38], and this anti-inflammation property may contribute to the neuroprotective effect in ischemic-reperfusion brain tissue damage [38]. In agreement, our data showed that pretreatment of PPAR-*β* agonist GW0742 doses-dependently reduced the level of NF-*κ*B as well as inflammatory cytokines TNF-*α*, IL-1*β*, and IL-6, while increased the level of IL-10 which, as an inhibitory cytokine, can inhibit the increase of IL-1, IL-6, and TNF-*α* [39, 40] and reduce cerebral ischemic injury [39, 40]. Combined with the fact that GW0742 largely improved the cytomorphological change of the hippocampus neurons by IRI, we reasonably presume that in our study, the improvement in GCIRI-induced memory and learning dysfunction by pre-activation of PPAR-*β* is, at least partially, a consequence of its anti-inflammatory properties in the hippocampus. 

Numerous studies have demonstrated that TNF-*α* and IL-1*β* induce NF-*κ*B activation synergically and regulate the expression of the downstream inflammatory cytokines such as iNO and COX2 through regulation of NF-*κ*B in the inflammatory response [41, 42], and NF-*κ*B activation in turn promotes TNF-*α* and IL-6 release [43, 44]. In other words, there is a positive feedback between NF-*κ*B and TNF-*α*/IL-6 with mutual promotion. Thus NF-*κ*B activation plays a proinflammatory effect [45, 46]. Other studies have shown that PPAR-*β* is an upstream effector molecule of NF-*κ*B and PPAR-*β* agonist can inhibit the increase of LPS-induced TNF-*α* and IL-1*β*-induced PGE_2_ by inhibiting NF-*κ*B activation [47, 48]. Therefore, the neuroprotective effect of GW0742 in the GCIRI may be associated with inhibition of the expression of various inflammatory factors such as TNF-*α*, IL-1*β*, and their downstream inflammatory cytokines by inhibition of NF-*κ*B activation and blocking the positive feedback between NF-*κ*B and TNF-*α*, IL-*β*.

## 5. Conclusions

In summary, we found that preactivation of PPAR-*β* could dose-dependently improve spatial memory and learning function and cytomorphological change of the hippocampus neurons after GCIRI. Moreover, we suggest that these effects are related to the anti-inflammation effect of PPAR-*β*, at least partly through inhibiting the expression and activation of NF-*κ*B in the hippocampus, and that PPAR-*β* may become a new therapeutic target for the treatment of GCIRI-induced impairment of memory and learning. 

## Figures and Tables

**Table 1:** Effect of GW0742 on the exploring time of rats after GCIRI.

Group	Exploring times (s)				
	Day 8	Day 9	Day 10	Day 11	Day 12
Sham group	159.70 ± 7.41	121.62 ± 8.18	75.48 ± 9.52	40.09 ± 5.82	20.67 ± 4.5
Model group	169.74 ± 6.87**	135.08 ± 7.09**	93.05 ± 8.97**	65.70 ± 7.40**	38.05 ± 7.28**
GW0742-20 group	170.57 ± 5.74	131.35 ± 7.24	88.86 ± 7.38	60.47 ± 6.88	34.28 ± 5.09
GW0742-60 group	166.40 ± 5.85	128.13 ± 6.77^#^	82.04 ± 7.83^##^	54.37 ± 7.05^##^	29.66 ± 6.21^##^
GW0742-180 group	163.69 ± 6.40^#^	125.46 ± 6.35^##^	78.31 ± 6.87^##^	48.67 ± 5.65^##/∆^	25.48 ± 5.83^##/∆^

Dates are expressed as mean ± SD (*n* = 12). **P < 0.01 compared with sham operation group; ^#^ *P* < 0.05, ^##^ *P* < 0.01 compared with model group; ^∆^ *P* < 0.05 compared with GW0742 20 group.

**Table 2:** Effect of GW0742 on the level of inflammatory factors in hippocampus of global cerebral ischemia/reperfusion rats.

Group	TNF-*α* (ng/mL)	IL-1*β* (pg/mL)	IL-6 (ng/mL)	IL-10 (ng/mL)
Sham group	2.24 ± 0.51	209.14 ± 48.29	5.90 ± 0.78	3.11 ± 0.26
Model group	6.51 ± 1.05**	545.14 ± 59.81**	13.39 ± 1.74**	1.97 ± 0.18**
GW0742-20 group	5.71 ± 0.98	495.09 ± 65.18	11.82 ± 1.33	2.09 ± 0.12
GW0742-60 group	5.08 ± 0.64^#^	452.20 ± 45.56^#^	9.87 ± 0.91^##^	2.28 ± 0.17^#^
GW0742-180 group	4.11 ± 0.58^##/∆^	369.99 ± 60.08^##/∆^	8.41 ± 1.06^##/∆^	2.72 ± 0.19^##/∆^

Dates are expressed as mean ± SD (*n* = 5). **P < 0.01 compared with sham operation group; ^#^ *P* < 0.05, ^##^ *P* < 0.01 compared with model group; ^∆^ *P* < 0.05 compared with GW0742 20 group.
